# IFN-γ-Primed hUCMSCs Significantly Reduced Inflammation *via* the Foxp3/ROR-γt/STAT3 Signaling Pathway in an Animal Model of Multiple Sclerosis

**DOI:** 10.3389/fimmu.2022.835345

**Published:** 2022-03-01

**Authors:** Xiao Ling, Teng Wang, Chao Han, Pin Wang, Xiaoli Liu, Chengyun Zheng, Jianzhong Bi, Xiaoyan Zhou

**Affiliations:** ^1^ Department of Gynaecology, the Second Hospital, Cheeloo College of Medicine, Shandong University, Jinan, China; ^2^ Department of Digestive Internal Medicine, the Second Hospital, Cheeloo College of Medicine, Shandong University, Jinan, China; ^3^ Department of Neurosurgery, the Second Hospital, Cheeloo College of Medicine, Shandong University, Jinan, China; ^4^ Department of Neurology Medicine, the Second Hospital, Cheeloo College of Medicine, Shandong University, Jinan, China; ^5^ Department of Hematology, the Second Hospital, Cheeloo College of Medicine, Shandong University, Jinan, China; ^6^ Institute of Biotherapy for Hematological Malignancies, Shandong University, Jinan, China; ^7^ Shandong University-Karolinska Institute Collaboration Laboratory for Stem Cell Research, Shandong University, Jinan, China

**Keywords:** IFN-γ-primed human umbilical cord mesenchymal stem cell (IFN-γ-hUCMSCs), multiple sclerosis (MS), experimental autoimmune encephalomyelitis (EAE), indoleamine 2,3-dioxygenease (IDO), Foxp3/ROR-γt/STAT3 signaling pathway

## Abstract

Our previous study showed that interferon gamma (IFN-γ) might enhance the immunosuppressive properties of mesenchymal stem cells (MSCs) by upregulating the expression of indoleamine 2,3-dioxygenease. Therefore, we treated experimental autoimmune encephalomyelitis (EAE) mice, an animal model of multiple sclerosis (MS), with IFN-γ-primed human umbilical cord MSCs (IFN-γ-hUCMSCs). This study aimed to investigate the potential therapeutic effects of IFN-γ-hUCMSCs transplantation and to identify the biological pathways involved in EAE mice. Firstly, the body weights and clinical scores of EAE mice were recorded before and after treatment. Then, the inflammatory cytokine levels in splenic cell supernatants were quantified by enzyme-linked immunosorbent assay. Finally, the mRNA expression levels of signal transducer and activator of transduction 3 (*STAT3*), retinoic acid-related orphan receptor gamma t (*ROR-γt*), and forkhead box P3 (*Foxp3*) were detected by quantitative reverse transcription polymerase chain reaction. We observed that IFN-γ-hUCMSCs transplantation significantly alleviated body weight loss and decreased the clinical scores of mice. Additionally, IFN-γ-hUCMSCs transplantation could regulate the production of inflammatory cytokines, interleukin (IL)-10 and IL-17, thereby showing more potent treatment efficacy than human umbilical cord MSCs (hUCMSCs) transplantation (*p* < 0.05). Compared with the EAE group, the expressions of *STAT3* and *ROR-γt* in the transplantation groups were significantly decreased, but the expression of *Foxp3* was significantly upregulated in the IFN-γ-hUCMSCs transplantation group compared to that in the hUCMSCs transplantation group. We assumed that IFN-γ-hUCMSCs may affect the balance of T helper 17 (Th17) cells/regulatory T cells (Tregs) through the Foxp3/ROR-γt/STAT3 signaling pathway to reduce the inflammatory response, thereby improving the clinical symptoms of EAE mice. Our study demonstrated that transplantation of IFN-γ-hUCMSCs could reduce inflammation in EAE mice *via* the Foxp3/ROR-γt/STAT3 signaling pathway, highlighting the therapeutic effects of IFN-γ-hUCMSCs in patients with MS.

## Introduction

Multiple sclerosis (MS) is a common cause of neurological disability among young people, resulting in increased economic and social costs (1, 2). MS has been investigated over a century, and although much has been discovered about the immunobiology, genetics, and epidemiology of this disease, the treatment outcomes remain unsatisfactory.

MS is characterized by inflammation-mediated demyelination of the white matter tracts with partial preservation of axons (3). Experimental autoimmune encephalomyelitis (EAE) is an animal model of MS that is particularly useful for testing new therapeutic approaches against MS (4, 5). Studies indicated that T helper (Th) cells, mainly Th1 and Th17 cells, which are characterized by the production of interleukin (IL)-17, are involved in the pathogenicity of MS and EAE, whereas regulatory T cells (Tregs) can maintain the autoimmune response (6, 7). One of the root causes of EAE is the decrease in the expression of forkhead box P3 (*Foxp3*)-expressing anti-autoimmune Tregs and an associated increase in autoimmune Th1 and Th17 cells (8). Moreover, Th17 cells, rather than Th1 cells, are important in autoimmune inflammatory conditions (9). Therefore, the balance of Th17/Tregs may play an important role in the modulation of EAE. IL-10 is an anti-inflammatory cytokine that plays a crucial role in preventing inflammatory and autoimmune pathologies and is involved in the regulation of the Janus kinase/signal transducers and activators of transduction (JAK/STAT) signaling pathway. Genetic deletion of *STAT3* in T cells has been shown to abrogate Th17 differentiation, suggesting that STAT3 is a potential therapeutic target for Th17-mediated diseases (10). Tregs can exert their functions by releasing inhibitory cytokines (IL-10 and IL-35) (11). Therefore, the production of inflammatory cytokines (IL-10 and IL-17) and the activation of the JAK/STAT signaling pathway were observed in our study.

With the development of novel treatment methods for autoimmune diseases, transplantation of mesenchymal stem cells (MSCs) has become popular as an effective strategy to counteract the progression of autoimmune disorders. Evidence suggests that MSCs can exert anti-inflammatory and immunomodulatory effects in various tissues, lower the clinical scores, and reduce central nervous system (CNS) leukocyte infiltration in EAE mice (12, 13). Our previous study showed that the immunosuppressive properties of MSCs may be enhanced by interferon gamma (IFN-γ) due to the upregulation of the tryptophan-catabolizing enzyme indoleamine 2,3-dioxygenease (IDO) (14). Therefore, we tried to treat EAE with IFN-γ-primed human umbilical cord MSCs (IFN-γ-hUCMSCs) and investigated their potential therapeutic effects on EAE mice.

## Materials and Methods

### Culture of hUCMSCs and IFN-γ-hUCMSCs

Human umbilical cord specimens were obtained from healthy Chinese young women who received cesarean section at the Second Hospital of Shandong University under sterile conditions. Informed consent was obtained from all participants before the collection of human umbilical cord specimens. This study was approved by the Ethics Committee of the Second Hospital of Shandong University, China.

The hUCMSCs were isolated and cultured, and the phenotype of the hUCMSCs was identified as described in our previous study (14). The umbilical cord was cut into small sections, following two washes with phosphate-buffered saline (PBS). The pieces were digested with collagenase for 1 h and trypsin-EDTA (Gibco, Waltham, MA, USA) for 30 min. The specimens were then filtered, the cells cultured in Dulbecco’s modified Eagle’s medium/nutrient mixture F-12 (HyClone, Logan, UT, USA), and were fixed with 10% defined fetal bovine serum (Gibco), epidermal growth factor, basic fibroblast growth factor (both from PeproTech, Cranbury, NJ, USA), l-glutamine (Gibco), and penicillin–streptomycin solution (HyClone) with 5% CO_2_ at 37°C for 72 h. After 3–5 days, non-adherent cells were removed and the medium was replenished. When the density of the cells reached 80%, they were digested with trypsin–EDTA at room temperature and passaged into new culture dishes. The expressions of the cell surface markers, including CD105, CD90, CD73, CD45, CD34, and HLA-DR (human leukocyte antigen—DR isotype), were evaluated using a LSRFortessa™ flow cytometer (BD Biosciences, Franklin Lakes, NJ, USA). Then, partial human umbilical cord MSCs (hUCMSCs) were pretreated with IFN-γ (20 ng/ml, 48 h). Cell suspensions were prepared at a density of 1 × 10^7^ cells/ml.

The whole process was performed in a Good Manufacturing Practice (GMP) laboratory.

### EAE Grouping and Cell Transplantation

Female C57BL/6J mice (6–8 weeks old; Biotechnology Co., Ltd., Beijing, China) were housed under a 12:12-h light/dark cycle in temperature- and humidity-controlled rooms. Mice were immunized with the myelin oligodendrocyte glycoprotein peptide 35-55 [MOG35-55; GL Biochem (Shanghai) Ltd., Shanghai, China] according to a previously published protocol (15). Priming mice to induce EAE involved complete Freund adjuvant (Sigma, St. Louis, MO, USA) containing 4 mg/ml *Mycobacterium tuberculosis* (strain H37Ra; Difco, Franklin Lakes, NJ, USA) and 200 μg MOG35-55. On days 0 and 2 post-immunization, 200 ng of pertussis toxin (Sigma) was administered intraperitoneally. Then, the mice were divided into three groups (*n* = 8 per group): the EAE group, the hUCMSCs transplantation (EAE+hUCMSCs) group, and the IFN-γ-hUCMSCs transplantation (EAE+IFN-γ-hUCMSCs) group. Fourteen days after immunization, each mouse in the transplantation groups was injected with 100 μl of the cell suspension (1 × 10^7^ cells/ml) *via* the tail vein. The mice in the EAE group were injected with 100 μl of PBS instead. All animal experiments were performed per the Ethical Committee for Animal Experiments of Shandong University, China.

### Body Weight and Clinical Score Assessment

After immunization, the mice were weighed and evaluated for signs of neurological disability. Clinical disability was scored as follows: 0, no paralysis; 1, loss of tail tone; 2, hindlimb weakness; 3, hindlimb paralysis; 4, hindlimb and forelimb paralysis; and 5, dying or dead (16). The scorers were blinded to the treatment groups.

### Splenocyte Culture and Detection of Inflammatory Cytokines

Two weeks after cell transplantation, the mice were anesthetized and sacrificed, and the spleens were removed. Splenocytes were cultured as described previously (17).

The spleen tissue was passed through a 100-μm nylon mesh screen to prepare single-cell suspensions, followed by removal of red blood cells with lysis buffer and incubation for 5 min. The suspensions were centrifuged (4°C at 1,200 rpm) and the supernatants collected. Splenocytes were washed twice with RPMI 1640 and adjusted to a density of 1 × 10^6^ cells/ml. The cells were incubated at 37°C for 2 h and then stimulated with MOG35-55 (15 μg/ml) for 48 h at 37°C in a 5% CO_2_ incubator. After 3 days, the supernatants were harvested and stored at −80°C for cytokine detection using enzyme-linked immunosorbent assay (ELISA).

The contents of IL-10 and IL-17 in the supernatants were assessed using commercially available ELISA kits (Proteintech, Shanghai, China) according to the manufacturer’s instructions.

### Real-Time Fluorescence Quantitative PCR

Total RNA from the lumbar myeloid tissue of mice was extracted using the TRIzol reagent (Life Technologies, Carlsbad, CA, USA) following the manufacturer’s instructions 2 weeks after cell transplantation. First-strand complementary DNA (cDNA) was synthesized using HiScript II Q RT SuperMix (Vazyme, Nanjing, China). Primers were supplied by Sangon Biotech (Shanghai, China). The primer sequences for *STAT3*, *ROR-γt*, and *Foxp3* are listed in **Table 1**. Quantitative real-time PCR (qRT-PCR) was performed with AceQ qPCR SYBR Green Master Mix (Vazyme) using the CFX96 Touch™ Real-Time PCR Detection System (Bio-Rad, Hercules, CA, USA). PCR was performed in triplicate, and all results were normalized to the expression of *GAPDH* using the 2^−ΔΔCt^ method.

**Table 1:** Primer sequences for quantitative real-time PCR (qRT-PCR).

Target gene	Primer sequences		Product length (bp)
*STAT3*	Forward	5′-GCCATCCTAAGCACAAAGC-3′	80
	Reverse	5′-GTGAAAGTGACCCCTCCTT-3′	
*ROR-γt*	Forward	5′-CTGACGGCCAACTTACTCTT-3′	150
	Reverse	5′-TGTCTGTCAGAGAGGCATATG-3′	
*Foxp3*	Forward	5′-GCATGTTCGCCTACTTCA-3′	242
	Reverse	5′-AGCCTCAGTCTCATGGTT-3′	
*GAPDH*	Forward	5′-CTGGGCTACACTGAGCACC-3′	101
	Reverse	5′-AAGTGGTCGTTGAGGGCAATG-3′	

### Statistical Analysis

Data were recorded separately and expressed as the mean ± standard deviation. Data were analyzed using one-way ANOVA and Tukey’s multiple comparisons test. Statistical significance was set at *p* < 0.05. All data were analyzed using SPSS software (version 23.0).

## Results

### Effects of hUCMSCs and IFN-γ-hUCMSCs Transplantations on the Body Weights and Clinical Scores of EAE Mice

**Figure 1:**
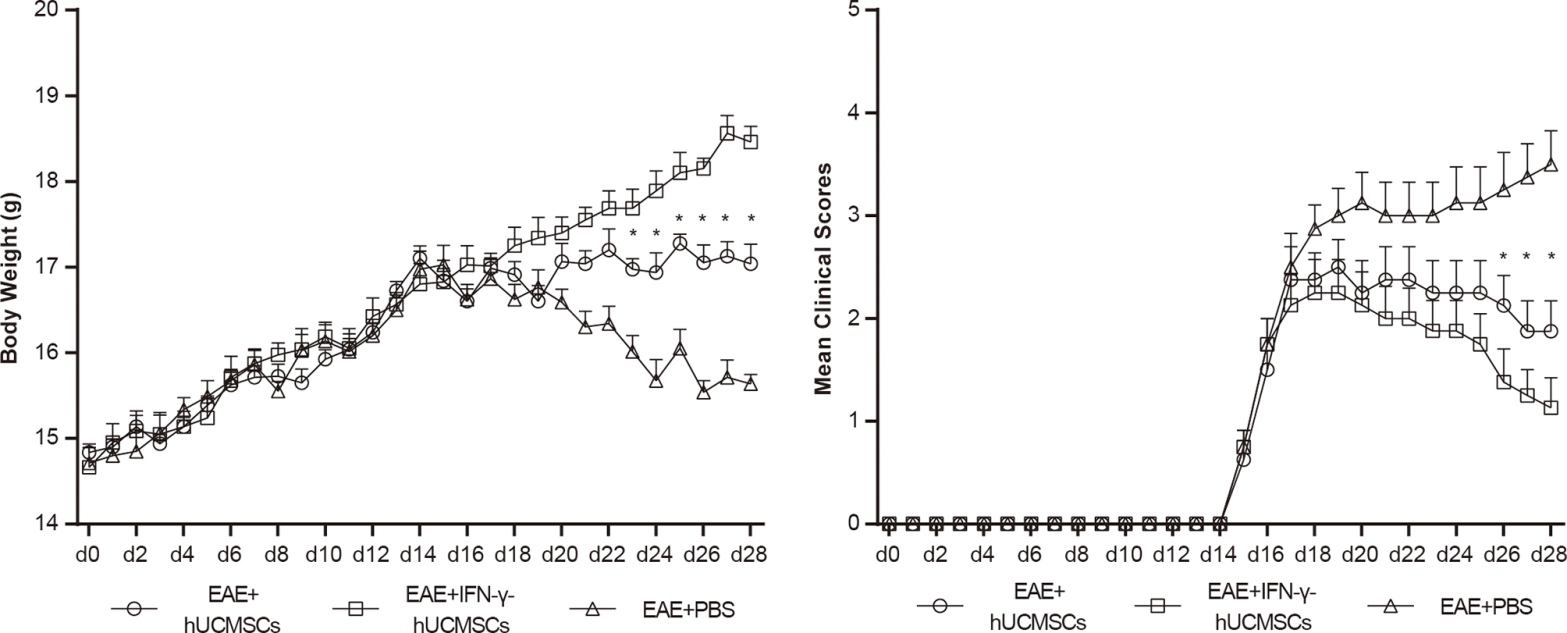
Effects of transplantation of human umbilical cord mesenchymal stem cells (hUCMSCs) and IFN-γ-primed hUCMSCs (IFN-γ-hUCMSCs) on the body weights and clinical scores of experimental autoimmune encephalomyelitis (EAE) mice. Transplantation of hUCMSCs and IFN-γ-hUCMSCs significantly alleviated the body weight loss and clinical scores of mice, especially in the IFN-γ-hUCMSCs transplantation group. *p < 0.05 (*n* = 8 per group).

The body weights and clinical scores of mice were recorded and assessed after immunization. After approximately 2 weeks, EAE mice began to lose weight, and the clinical scores started to increase. We observed that transplantation of hUCMSCs and IFN-γ-hUCMSCs significantly alleviated body weight loss and decreased the clinical scores of mice, especially in the IFN-γ-hUCMSCs transplantation group (**Figure 1**).

### Inflammatory Cytokines of Splenocyte Culture Supernatants

**Figure 2:**
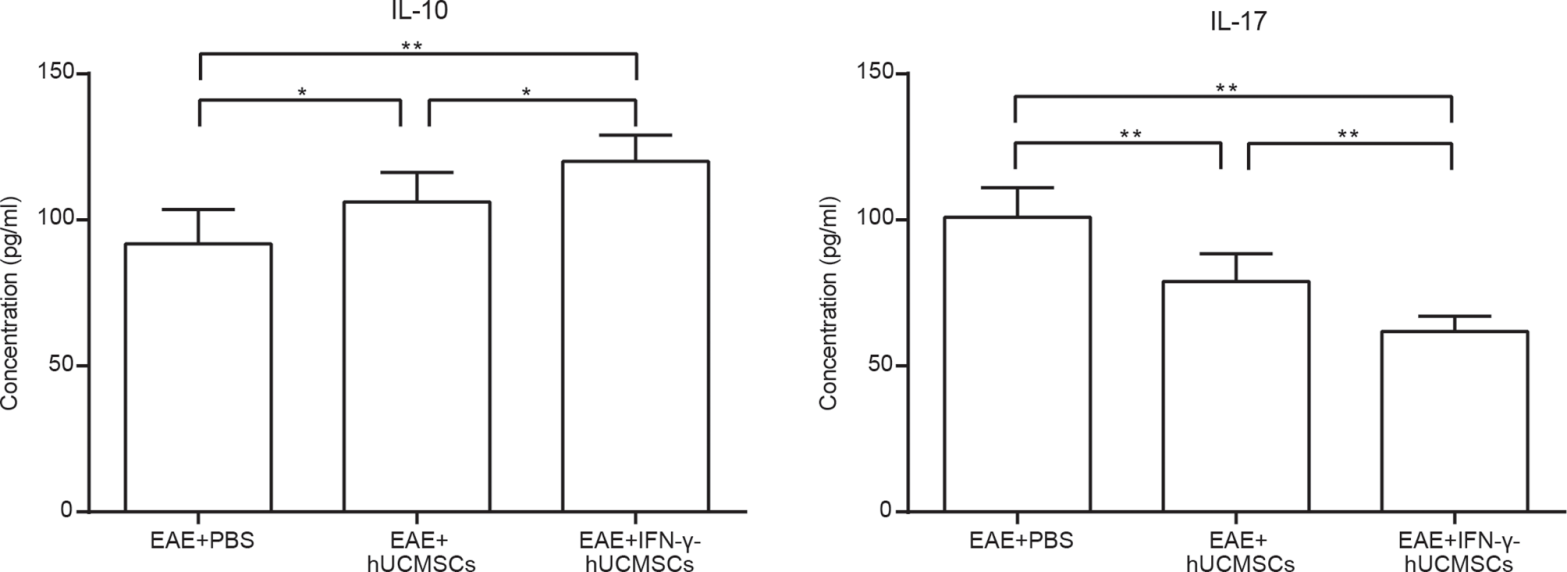
Effects of transplantation of human umbilical cord mesenchymal stem cells (hUCMSCs) and IFN-γ-primed hUCMSCs (IFN-γ-hUCMSCs) on the levels of IL-10 and IL-17 in experimental autoimmune encephalomyelitis (EAE) mice. Cell transplantation could increase the concentration of IL-10 and reduce the concentration of IL-17, especially in the IFN-γ-hUCMSCs transplantation group. *p < 0.05, **p < 0.01 (*n* = 8 per group).

To investigate the changes in the inflammatory cytokines after cell transplantations, the contents of IL-10 and IL-17 in splenocyte culture supernatants were tested using ELISA. We discovered that transplantation of hUCMSCs and IFN-γ-hUCMSCs could increase the concentration of IL-10, especially in the IFN-γ-hUCMSCs group. Conversely, IL-17, the pro-inflammatory cytokine, was observed to be remarkably lower in both cell transplantation groups, especially in the IFN-γ-hUCMSCs group (**Figure 2**). The results showed that transplantation of IFN-γ-hUCMSCs could regulate the inflammation response more effectively than hUCMSCs in EAE mice.

### mRNA Expression Levels of *Foxp3*, *ROR-γt*, and *STAT3*

**Figure 3:**
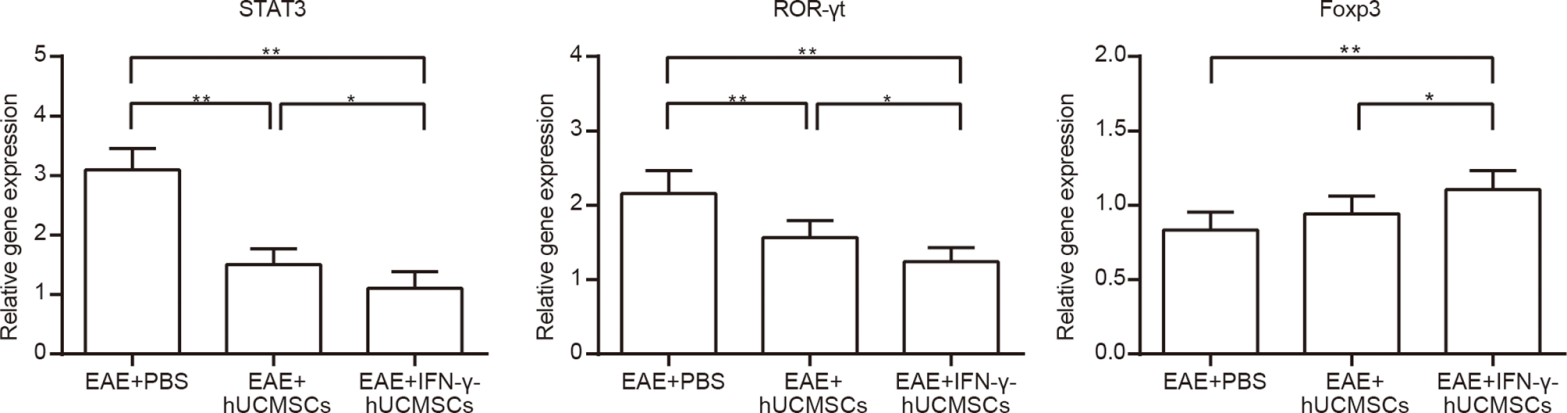
Messenger RNA (mRNA) expressions of *Foxp3*, *ROR-γt*, and *STAT3* in the cell transplantation groups. The expressions of *STAT3* and *ROR-γt* were significantly downregulated and that of *Foxp3* was upregulated in the cell transplantation groups, especially in the IFN-γ-primed human umbilical cord mesenchymal stem cells (IFN-γ-hUCMSCs) transplantation group. *p < 0.05, **p < 0.01, (*n* = 8 per group).

The messenger RNA (mRNA) expression levels of *Foxp3*, *ROR-γt*, and *STAT3* were determined using qRT-PCR in all transplantation groups (**Figure 3**). Compared with those in the EAE group, the expressions of *STAT3* and *ROR-γt* in the cell transplantation groups were significantly decreased, but the expression of *Foxp3* was upregulated. However, the increase in *Foxp3* expression was more significant in the IFN-γ-hUCMSCs transplantation group than that in the hUCMSCs transplantation group.

## Discussion

MS is a demyelinating disorder induced by activation of the autoimmune system. The treatments for MS mainly include the administration of glucocorticoids in the acute phase, disease modification therapy (DMT) to reduce the inflammatory activity and improve long-term prognosis, and symptomatic treatment of complications; however, the treatment outcomes are not satisfactory. Therefore, it is important to discover effective therapeutic strategies for patients with MS. MSCs have shown strong immunomodulatory functions in many diseases, especially autoimmune diseases (18). Therefore, transplantation of MSCs is a promising therapeutic strategy for patients with MS.

Studies have shown that MSCs may suppress the activity of B cells and reduce autoantibody production (18). In addition, they can promote the expansion of Tregs by secreting IL-10, IDO, and transforming growth factor beta (TGF-β) (19, 20). Moreover, MSCs can suppress the differentiation and proliferation of Th cells and increase the suppressive proportion of B cells *via* IDO (21, 22), and IFN-γ is the main inducer of IDO (23). IFN-γ-primed MSCs (IFN-γ-primed MSCs) showed stronger ability to regulate the inflammation and immune suppression than MSCs *in vitro* (24). Therefore, we investigated the effects of IFN-γ-hUCMSCs on EAE mice established for MS studies.

In our study, we observed that IFN-γ-hUCMSCs transplantation significantly alleviated body weight loss and decreased the clinical scores of mice. In addition, IFN-γ-hUCMSCs transplantation could regulate the production of inflammatory cytokines (IL-10 and IL-17), thereby showing significantly higher potent treatment efficacy than hUCMSCs transplantation. IL-10, along with its receptors, plays an important role in the pathogenesis of various diseases, including infectious, inflammatory, and autoimmune diseases (25, 26). Studies have shown that bone marrow-derived MSCs can inhibit Th17 cell differentiation *via* IL-10 secretion (27). IL-17 is a signature cytokine of Th17 cells. The orphan nuclear receptor ROR-γt is the master regulator that drives the differentiation of Th17 cells (28). Recent evidence has shown that ROR-γt can potently upregulate IL-17 reporter activity without the involvement of any other factors (29). Furthermore, IL-10 may suppress the expression of *ROR-γt.* Foxp3^+^ Tregs are a special lineage of cells central to the maintenance of immunological tolerance (11, 30). They function as transcriptional repressors for various transcription factors. Foxp3 directly interacts with ROR-γt through the exon 2 region of *Foxp3* (29). STAT3 signaling is essential for the induction of ROR-γt and the subsequent Th17 cell differentiation, and the SRY-related high-mobility group (HMG) box5 (Sox5) and c-Maf may cooperatively induce Th17 cell differentiation *via* the induction of ROR-γt as downstream targets of STAT3 (31, 32). Therefore, we speculated whether the changes in the levels of inflammatory cytokines (IL-10 and IL-17) were related to the mRNA expressions of *ROR-γt*, *Foxp3*, and *STAT3.*

As mentioned above, the imbalance of Th17/Tregs is involved in the pathogenesis of EAE. STAT3 and ROR-γt can promote the differentiation of Th17 cells. Foxp3 is necessary for Tregs to exert immunosuppressive function and is negatively regulated by STAT3. Genetic deletion of *STAT3* in T cells has been shown to abrogate Th17 differentiation, suggesting that the inhibition of STAT3 can tilt the balance of Th17/Tregs toward Tregs. Combined with the results of our study, we observed that, compared to those in the EAE group, the expressions of *STAT3* and *ROR-γt* in the transplantation groups were significantly decreased, but the expression of *Foxp3* was upregulated, especially in the IFN-γ-hUCMSCs transplantation group. We assumed that IFN-γ-hUCMSCs may affect the Th17/Tregs balance through the Foxp3/ROR-γt/STAT3 signaling pathway to reduce the inflammatory response, thereby improving the clinical symptoms in EAE mice. This study has some possible limitations. The inhibition experiment should be studied in the future.

In summary, we found that IFN-γ-hUCMSCs could significantly alleviate the body weight loss and clinical scores of EAE mice and regulate the production of inflammatory cytokines *via* the Foxp3/ROR-γt/STAT3 signaling pathway.

## Conclusion

Our study demonstrated that transplantation of IFN-γ-hUCMSCs could reduce inflammation in EAE mice *via* the Foxp3/ROR-γt/STAT3 signaling pathway, which highlights the therapeutic effects of IFN-γ-hUCMSCs in patients with MS. These results suggest that transplantation of IFN-γ-hUCMSCs may be a potential therapy for MS.

## Data Availability Statement

The raw data supporting the conclusions of this article will be made available by the authors, without undue reservation.

## Ethics Statement

The studies involving human participants were reviewed and approved by the Ethics Committee of the Second Hospital of Shandong University. The patients/participants provided written informed consent to participate in this study. The animal study was reviewed and approved by Ethics Committee of the Second Hospital of Shandong University.

## Author Contributions

All authors listed have made a substantial, direct, and intellectual contribution to the work and approved it for publication.

## Funding

This work was supported by the National Natural Science Foundation of China (81870848), the Fundamental Research Funds of Chinese Academy of Medical Sciences(2019-RC-HL-026), Shandong University Multidisciplinary Research and Innovation Team of Young Scholars(2020QNQT019).

## Conflict of Interest

The authors declare that the research was conducted in the absence of any commercial or financial relationships that could be construed as a potential conflict of interest.

## Publisher’s Note

All claims expressed in this article are solely those of the authors and do not necessarily represent those of their affiliated organizations, or those of the publisher, the editors and the reviewers. Any product that may be evaluated in this article, or claim that may be made by its manufacturer, is not guaranteed or endorsed by the publisher.
